# Sintero server scalable interoperability framework for DALLAS communities

**Published:** 2012-06-15

**Authors:** Ed Conley, Peter Burnap, Tim Benson, Ian Taylor, Andrew Harrison, Philip Scott, Sasha Karakusevic

**Affiliations:** Cardiff University, UK; Cardiff University, UK; Cardiff University, UK; Cardiff University, UK; Cardiff University, UK; University of Portsmouth, UK; University of Bristol, UK

**Keywords:** DALLAS, interoperability, integrated care

## Abstract

We describe a prototype open source UK-scalable Health Information Exchange (HIE) to support patient-centric care, translational research and other secondary uses. It has been designed by interoperability experts to standardise information flows across the patient path—for example from home, work, mobile, clinical and community care locations. Sintero enables secure information sharing within the patient’s named ‘circle of care’ including family, third sector, social care, community pharmacy, specialist care networks and named statutory (e.g. NHS) carers. Patients may access to their own personal health records and manage consents for their sharing and secondary uses. Sintero’s infrastructure is a low-cost ‘commodity’ approach to delivering scalable DALLAS community solutions. It does this by taking a collaborative approach to capacity development, working with NHS informatics and frontline innovation centres towards efficient use of the technology by the workforce. There is a strong focus on delivery of patient value by continuous measurement and systematic feedback of outcomes. Sintero is being made available to UK DALLAS communities on a collaborative basis.

